# PReventing and Approaching Crises for frail community-dwelling patients Through Innovative Care (PRACTIC): protocol for an effectiveness cluster randomised controlled trial

**DOI:** 10.1186/s13063-024-08117-6

**Published:** 2024-05-06

**Authors:** Anette Væringstad, Ellen Thea Gjelseth Dalbak, Daniela Holle, Janne Myhre, Øyvind Kirkevold, Sverre Bergh, Bjørn Lichtwarck

**Affiliations:** 1 The Research Centre for Age-Related Functional Decline and Disease, Innlandet Hospital Trust, Ottestad, Norway; 2 Department of Health, Care and Nursing, Faculty of Medicine NTNU, Norwegian University of Science and Technology, Gjøvik, Norway; 3 Department of Nursing Science, University of Applied Sciences (HS Gesundheit), Bochum, Germany; 4 Faculty of Social and Health Sciences, Inland Norway University of Applied Sciences (INN University), Elverum, Norway; 5 The Norwegian National Centre for Ageing and Health, Vestfold Hospital Trust, Vestfold, Norway

**Keywords:** Frailty, Crises, Home care services, Community-dwelling people, Psychosocial interventions, Case conferences, Participatory action research (PAR), Randomised controlled trial (RCT), PRACTIC Goal Setting Interview (PGSI)

## Abstract

**Background:**

Demographic changes, with an increasing number and proportion of older people with multimorbidity and frailty, will put more pressure on home care services in municipalities. Frail multimorbid people receiving home care services are at high risk of developing crises, defined as critical challenges and symptoms, which demand immediate and new actions. The crises often result in adverse events, coercive measures, and acute institutionalisation. There is a lack of evidence-based interventions to prevent and resolve crises in community settings.

**Methods:**

This is a participatory action research design (PAR) in a 6-month cluster randomised controlled trial (RCT). The trial will be conducted in 30 municipalities, including 150 frail community-dwelling participants receiving home care services judged by the services to be at risk of developing crisis. Each municipality (cluster) will be randomised to receive either the locally adapted TIME intervention (the intervention group) or care as usual (the control group). The Targeted Interdisciplinary Model for Evaluation and Treatment of Neuropsychiatric Symptoms (TIME) is a manual-based, multicomponent programme that includes a rigorous assessment of the crisis, one or more interdisciplinary case conferences, and the testing and evaluation of customised treatment measures. PAR in combination with an RCT will enhance adaptations of the intervention to the local context and needs. The primary outcome is as follows: difference in change between the intervention and control groups in individual goal achievement to resolve or reduce the challenges of the crises between baseline and 3 months using the PRACTIC Goal Setting Interview (PGSI). Among the secondary outcomes are the difference in change in the PGSI scale at 6 months and in neuropsychiatric symptoms (NPSs), quality of life, distress perceived by professional carers and next of kin, and institutionalisation at 3 and 6 months.

**Discussion:**

Through customised interventions that involve patients, the next of kin, the social context, and health care services, crises may be prevented and resolved. The PReventing and Approaching Crises for frail community-dwelling patients Through Innovative Care (PRACTIC) study will enhance innovation for health professionals, management, and users in the development of new knowledge and a new adapted approach towards crises.

**Trial registration:**

ClinicalTrials.gov identifier: NCT05651659. Registered 15.12.22.

**Supplementary Information:**

The online version contains supplementary material available at 10.1186/s13063-024-08117-6.

## Administrative information

Note: the numbers in curly brackets in this protocol refer to SPIRIT checklist item numbers. The order of the items has been modified to group similar items (see http://www.equator-network.org/reporting-guidelines/spirit-2013-statement-defining-standard-protocol-items-for-clinical-trials/).

**Table:** 

Title {1}	Preventing and approaching crises for frail community-dwelling patients through innovative care (PRACTIC): Protocol for an effectiveness cluster randomised controlled trial
Trial registration {2a and 2b}.	ClinicalTrials.gov identifier, ID: NCT05651659. Registered the 15th of December, 2022. https://register.clinicaltrials.gov/prs/app/template/Preview.vm?epmode=View&popup=true&uid=U0006CJN&ts=58&sid=S000C7YR&cx=3tkuqm
Protocol version {3}	Version 9, 28.11.23.
Funding {4}	The study is funded in total by a grant from the Innlandet Hospital Trust, grant number 150667.
Author details {5a}	1 The Research Centre for Age-related Functional Decline and Disease, Innlandet Hospital Trust, Ottestad, Norway. 2 Department of Health, Care and Nursing, Faculty of Medicine NTNU, Norwegian University of Science and Technology, Gjøvik, Norway. 3 Department of Nursing Science, University of Applied Sciences (HS Gesundheit), Germany. 4 Faculty of Social and Health Sciences, Inland Norway University of Applied Sciences, Elverum, Norway (INN University). 5 The Norwegian National Centre for Ageing and Health, Vestfold Hospital Trust, Vestfold, Norway.
Name and contact information for the trial sponsor {5b}	Sykehuset Innlandet, HF, Postboks 104, 2381 Brumunddal
Role of sponsor {5c}	The funding body did not take part in the design of the study, data collection, analysis, interpretation of data or in the writing of the manuscript..

## Introduction

### Background and rationale {6a}

Worldwide, the proportion and absolute number of older individuals are increasing dramatically. The population aged 60 and older is expected to double by 2050 worldwide, and the proportion of people receiving care at home has increased over the past 10 years [1, 2]. In Norway, approximately 200,000 people are currently receiving home care services, while there are approximately 40,000 beds in nursing homes [3]. The majority of people would rather reside and receive care at their homes than in an institution [4]. One of the main health and societal challenges for municipalities, now and in the future, is to offer high-quality health care services for a growing population of older people with complex needs due to frailty and multimorbidity [5]. Patients may experience distress because they are unable to manage the situation at home, even though they may prefer to avoid institutionalisation. The considerable distress experienced by informal caregivers revolves around the lack of sufficient support from home care services and the limited availability of nursing home places [6]. Home care services can be described as a complex organisation with various service components ranging from practical assistance in the home to the delivery of advanced medical treatment. To enable people to live safely at home, home care services are interdependent on other sectors, such as general practitioners (GPs), the hospital sector, primary health care workers, and social care [4, 7].

There is a significant variation among patients receiving home care services in terms of functional abilities, age, living conditions, and chronic diseases [8]. A considerable number of these patients have multiple chronic conditions, commonly referred to as multimorbidity [4]. Estimates suggest that within the next 20 years, the population of elderly individuals with multimorbidity will double [9]. The prevalence of frailty is estimated to be 11% among adults aged ≥ 65 years, increasing to 50% among those > 80 years of age [10]. Most frail individuals are multimorbid, but not all multimorbid individuals are frail [11]. Frailty has been described as a state of physiological vulnerability with a reduced capacity to adapt and manage internal and external stressors [12]. Studies have emphasised the relevance and utility of a biopsychosocial definition of frailty, including the terms physical frailty, psychological frailty, and social frailty. Social frailty is an important dimension of the frailty concept and makes people with low incomes, low educational levels, and low housing standards vulnerable to various adverse health outcomes. Combining these three dimensions into a multidimensional concept of frailty promotes the use of targeted multidomain interventions tailored to older adults’ frailty status [13, 14].

### Definition of crisis

People who are at an increased risk of developing crises are often frail. Crises are major stressors for patients, their next of kin, and the care staff and often lead to adverse events, acute institutionalisation, and the use of coercion [15, 16]. Crises can be described as ‘a process in which the stressors cause an imbalance requiring an immediate decision which leads to a desired outcome and therefore crisis resolution’ [15]. In the Preventing and Approaching Crises for Frail Community-dwelling Patients Through Innovative Care (PRACTIC) study, we will operationalise this definition to describe crises in practice as ‘critical challenges and symptoms that demand immediate and new actions’. The challenges and symptoms that trigger and maintain crises are heterogeneous and vary between patients and may include depression, poor nutrition status, rejection of care, incontinence, neuropsychiatric symptoms (NPSs), and social isolation [12]. The ‘Mind the Gap report’ from the Advisory Board of the Global Forum for Health Care Innovators states that 1–5% of community-dwelling patients are high-risk patients and 15–35% are patients with an increasing risk. The literature on crises among patients receiving home care services has mainly explored the phenomenon in relation to people with dementia living at home [15, 16].

One of the most demanding challenges for health care authorities and home care services is to develop and implement high-quality health care models for the growing population of frail community-dwelling patients [17]. In addition to early recognition and response to clinical signs and symptoms, as recommended by the Norwegian Health Directorate, providing health care for this group of frail patients represents a change from a merely task-oriented service to a service that aims to assess the complex biopsychosocial character of frailty [18]. There are large variations in the content and organisation of Norwegian home care services, and research pertaining to these services has largely been descriptive, with a preponderance of qualitative studies [19, 20]. There is a paucity of studies investigating the effectiveness of interventions [20]. A study testing a structured follow-up programme using a checklist for frail community-dwelling adults found no common perception among nurses or their leaders that the approach was useful to ensure high-quality health care [21]. This finding supports the conclusion of a Cochrane Review in 2016 summarising primary care interventions for patients with multimorbidity [22]. The review revealed no clear positive improvements in clinical outcomes, health service use, medication adherence, patient-related health behaviours, health professional behaviours, or costs. The authors concluded that to improve outcomes for people with multiple conditions, there is a need for new multicomponent interventions, targeting both the heterogeneity of patients and their multimorbidity. To our knowledge, no effectiveness study of interventions targeting heterogeneous groups of home-dwelling patients with multimorbidity has been conducted in Norway. The proposed project will develop knowledge beyond the current state by also including the experimental testing of an intervention [20].

Participatory action research (PAR) in combination with an RCT has been suggested as a design to enhance local adaptations of an intervention to the local context and needs [23, 24]. There are multiple variations in the content and organisation of Norwegian home care services, with various service components ranging from practical assistance in the home to the delivery of advanced medical treatment [19]. The possibility of success for innovative interventions is probably higher if the interventions are not too complex, with no aim of changing the organisation of health care services [23, 25]. A flexible complex intervention has been emphasised as an important factor for interventions to be effective and increasing their applicability [24, 26–28]. According to Hawe et al. [24], it is the function and processes of the intervention that should be standardised, not the components in the intervention. Adaptation of the components should be performed both at the research project level and at the implementation level in municipalities [24]. Using a PAR design will help to adapt the components of the intervention to these variations, thereby enhancing implementation in each municipality. As a part of the main PRACTIC study, a process evaluation study will be conducted in parallel with the RCT [29]. This will ensure that these variations in the organisation mentioned earlier and the necessary adaptations are accounted for.

### The TIME intervention

TIME (Targeted Interdisciplinary Model for Evaluation and Treatment of Neuropsychiatric Symptoms) is a Norwegian evidence-based model for problem-solving regarding neuropsychiatric symptoms (NPSs) in dementia and other mental diseases. The model is based on the theoretical frameworks of cognitive behavioural therapy (CBT) and person-centred care (PCC) [28]. TIME has also been used in clinical practice for other complex issues, such as nutritional failure, multimorbidity, and general functional loss [30]. It is a multicomponent interdisciplinary model consisting of three overlapping phases, which are the core components of the model. First is the assessment phase where the care staff and the physician collaborate in a comprehensive biopsychosocial assessment. The second phase is the reflection phase with interdisciplinary case conferences based on principles from cognitive behavioural therapy (the ABC method), where a customised treatment plan is developed. The ABC method from cognitive behavioural therapy is used as an analytic tool for the analyses of complex challenges in case conferences [31]. The third phase is the action and evaluation phase, and each treatment measure in the plan is implemented and systematically evaluated. The TIME model is effective for treating NPSs in dementia and has been proven feasible in nursing homes (NHs) [28, 32]. Our research centre has pilot tested the model in home care services [33]. One of the assets of TIME is interdisciplinary case conferences, and interdisciplinarity is essential in the approach to a crisis. Based on the results from the pilot test, the inclusion criteria for the patients were broadened, and the content of the training for all employees was further developed [33]. In addition, the schedule for the training and implementation was adapted to everyday routines for home care services. Further adaptation of the TIME model to home care services will be performed continuously at local project group meetings during the RCT in the intervention municipalities.

### Theoretical framework

The intervention with TIME is based on a theoretical framework of complexity science and a biopsychosocial understanding of crises [34, 35]. In this framework, frailty means that the frail patient is prone to instability caused by complex interactions among biological, psychological and social stressors [14, 35]. If this instability rises, it eventually culminates in the development of a crisis that, according to our description of crises, demands immediate and new actions. Describing frailty and crises as complex phenomena sets the stage for why interventions should be constructed and implemented as flexible complex interventions to be able to assess, prevent, and resolve crises [24]. This also includes the choice of design and methods for testing the effectiveness of an intervention.

## Objectives {7}

### Aims and hypothesis

The primary purpose of this study is to test the effectiveness of an adapted version of a biopsychosocial person-centred model (TIME) to prevent and resolve crises for frail community-dwelling people receiving home care services. We hypothesise that the adapted version of the TIME intervention would prevent and resolve crises compared to a control condition consisting of usual care.

### Trial design {8}

**Fig. 1:**
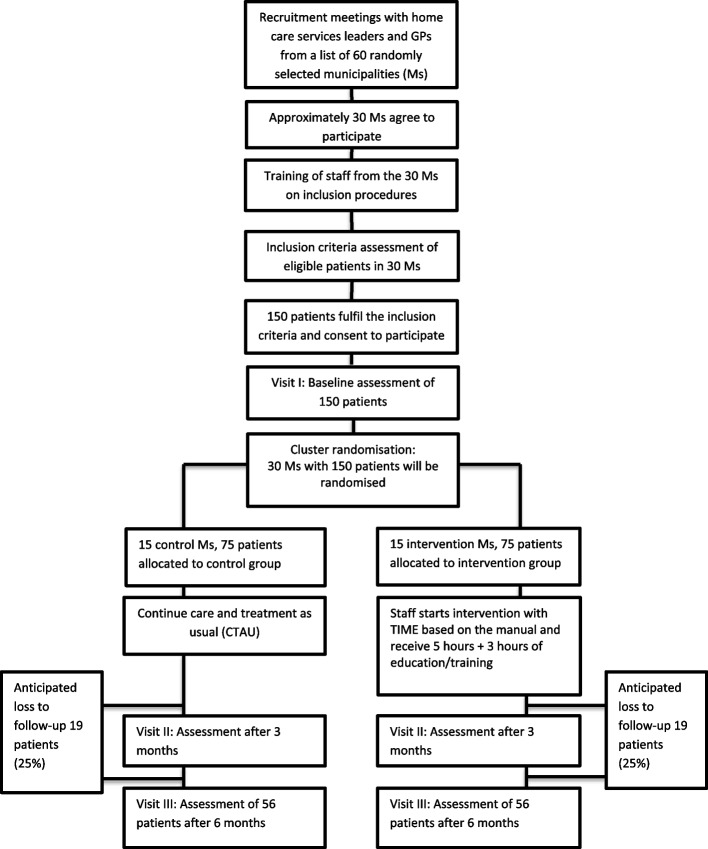
The PRACTIC trial: flowchart of the clusters and patients throughout the phases of the trial

This is a pragmatic study (effectiveness study) focusing on testing the effect of an intervention under real-world conditions. We will use a participatory action research (PAR) design in a cluster randomised controlled trial (RCT) with two parallel groups: intervention municipalities (IMs) and control municipalities (CMs) [23, 36]. Figure 1 shows a flow chart of the clusters and individuals throughout the phases of the trial based on the power calculation.

PAR aims to ensure adaptation of the components of the intervention to the local context [23, 36]. This will be done by establishing a local project team in each municipality consisting of two representatives from the research group and local participants (representatives of local managers, staff, and GPs). These groups will adapt the implementation process and the TIME model according to the cyclic Deming process: plan, do, check, act, and adjust [37]. The implementation process will be adapted to the local context to fit with the organisational structure already established in home care services. For example, the content and time used for educational and training purposes can vary depending on the educational level and established educational arenas. These variations will be registered during the process. For the TIME model, the core components (functional) of the intervention (TIME) must be fixed, and other components can be adapted to the local context and the patients included. An assessment phase must be performed, but *what* to assess and the *types* of clinical scales to be used can vary (processes). Case conferences using the inductive cognitive ABC model for the analyses of crises must be conducted, but the timeframe and participants of these conferences will vary. The action and evaluation phase must be conducted, but the timeframe and types of actions and evaluations can vary. These adaptations of the model will be accurately mapped in each setting during the study, and this mapping will be a part of the process evaluation study in the PRACTIC study [29]. In this trial, we will follow the recommendations from the CONSORT statement for randomised trials of nonpharmacologic treatments [38].

## Methods: participants, interventions, and outcomes

### Study setting {9}

This trial is part of the larger PRACTIC (Preventing and Approaching Crises for Frail Community-dwelling Patients Through Innovative Care) study. The trial will include approximately 30 randomly selected municipalities and their home care services from all health care regions in Norway. From each region, a sample of small, medium, and large municipalities will be invited to participate. From these 30 included municipalities, 150 users of home care services and their next of kin will be invited to participate in the trial.

### Eligibility criteria {10}

The inclusion criteria for patients are as follows: (1) in need of home care services, (2) a score ≥ 5 on the Clinical Frailty Scale (indicating mild to severe frailty) [39], and (3) perceived by the home care service as being in an unstable situation with a high risk for acute institutionalisation or showing resistance to care. The only exclusion criterion is an expected short life expectancy (i.e. < 4 weeks).

### Who will take informed consent? {26a}

Approximately four project nurses from each home care service unit in the municipality will be trained to obtain informed consent. For more details of the recruitment of these project nurses and the training session, see the “Intervention description {11a}” section.

### Additional consent provisions for collection and use of participant data and biological specimens {26b}

N/A. Participant data and biological samples are not collected in ancillary studies.

## Interventions

### Explanation for the choice of comparators {6b}

#### A goal-oriented primary outcome

To evaluate the effects of a biopsychosocial intervention to prevent and resolve crises in a heterogeneous population, there is a need for a goal-oriented outcome comprising this variability. The goal of the intervention and the outcome will necessarily vary from patient to patient [26]. We have therefore translated and modified a validated individual goal-oriented interview (The Bangor Goal-Setting Interview, BGSI) [40] to establish a common primary outcome to be used in the RCT. The PRACTIC Goal Setting Interview (PGSI) is a Norwegian adapted version of the BGSI. In the PGSI, the individual goals set for each patient represent treatment and actions targeting the challenges and symptoms that trigger and maintain the patient’s crisis. The difference in goal achievement between the intervention and control groups, as further explained in the “Methods” section, defines our primary outcome.

In this trial, the primary outcome is the difference in change between the intervention and control groups in individual goal achievements assessed by the PGSI [39]. The patients in the control group will receive care and treatment as usual, but they will probably also profit from the extra attention given by the home care services because of their participation in the RCT and because of the use of the measure for the primary outcome, the PGSI. In this way, we can isolate any effects on goal achievements to prevent or resolve crises to the main difference between IMs and CMs, i.e. the TIME intervention.

### Intervention description {11a}

#### Joint education and training for the staff in intervention municipalities (IMs) and control municipalities (CMs)

Depending on the size and organisation of the home care services, approximately four project nurses from each organisational unit of the home care services in each IM and CM will be given special responsibility in the trial. Before randomisation, these nurses will complete a 1-day educational course on the procedures for the trial. Their main task will be to recruit participants according to the inclusion criteria, obtain written consent for participation, and facilitate the interviews for the assessments of the participants at baseline, 3 months, and 6 months. The manager of the home care services will select these nurses in the municipality based on the following criteria: health care professionals who work on a nearly full-time basis, have shown interest in professional development, and have gained legitimacy with the rest of the staff. Thus, these health care professionals can be selected among registered nurses, auxiliary nurses, or members of other professional groups (e.g. social workers or occupational therapists) in home care services.

After this coeducational session for both the IMs and CMs, the CMs will continue care and treatment as usual (CTAU). Care and treatment as usual will usually involve medication follow-up and medical procedures, personal care, dressing, and bathroom assistance.

#### Specific education and training of staff in the IMs

The staff in the IMs will complete 4 h of lectures, training, and role-play related to TIME. The educational programme is aimed at as many employees as possible in the organisation and provides basic knowledge about the TIME model. The education and training team will consist of eight specialist registered health care professionals in geriatrics or geriatric psychiatry and one physician with special competence in nursing home medicine. All members of the education and training team are familiar with TIME and have used the model for some years in real-world clinical settings. The lectures will be standardised according to the steps listed in the TIME manual.

The leaders of the home care service in the IMs will attend these lectures to ensure that these leaders provide support to the staff during the trial. We will also encourage the GPs in the municipalities to participate. Each staff member in the IMs will be provided with the TIME manual, which describes the intervention step by step. They will also be given access to an educational film about TIME and to a website to support the intervention. The project nurses from the IMs who participated in the joint education and training for the inclusion criteria will now hold a special responsibility for putting the model into practice based on the manual. These nurses will therefore receive three additional hours of education, training, and role-play on the different components of TIME and the implementation of the intervention. In the trial, they will be referred to as TIME administrators. Immediately after randomisation and allocation, the project management team will contact these TIME administrators via telephone and instruct them to begin to implement the intervention according to the TIME manual for the patients included in the trial. This telephone call will be made from a few days up to 1 week before the education and training sessions are given. The TIME manual is available online.

The TIME model is described in detail in the TIME manual, which provides a step-by-step guide for implementing the model [24]. The different components of TIME acting together thus produce a standardised approach with the goal of preventing and resolving crises for patients receiving home care services. In the assessment phase, the GP will carry out a somatic and mental examination of the patient, and medicine prescriptions will be critically reviewed. The staff will gather the patient’s background information and medical history, assess the level of activities of daily living [41] and nutritional status (the Malnutrition Screening Tool, MST) [42], and participate in the goal-setting interview (PGSI). This phase is described in Table 1. The duration of this phase varies from 1 day to 4 weeks, depending on the patient’s situation and symptoms. After the assessment phase, the reflection phase begins. At this stage, a case conference is held for all stakeholders around the patient, i.e. nursing staff, GPs, physiotherapists, and occupational therapists. The aim of the case conferences is to create a mutual understanding of the patient’s situation and to tailor a detailed treatment plan to be tested in the coming weeks. This reflection is carried out systematically using a five-column table on a board or a screen, and the following five aspects are reviewed: considered facts, interpretation, feelings within the staff about the situation, actions to take, and evaluation. The timeframe and agenda for the case conferences are outlined in Table 2. The final phase is the action and evaluation phase. In this phase, each treatment measure in the plan is put into practice and then systematically evaluated. The timeframe for the entire intervention with TIME will vary from 1 or 2 weeks up to 8 weeks depending on the patient’s situation.

**Table 1:** The assessment phase

**Checklist for the assessment phase**	
The following should be performed:	PRACTIC Goal Setting Interview (PGSI)
	Personal history and conversation with the person: what is the person’s perspective? For example, “Who am I?”
	Medical history: A summary
	Somatic and psychological assessment and examination
	ADL assessment: Activities of daily living Nutritional screening
Suspected conditions:	☐Yes ☐No If dementia is suspected, start a basic dementia assessment. In the case of known dementia, assess the degree of dementia
	☐Yes ☐No If pain is suspected, conduct a pain assessment
	☐Yes ☐No In the case of behavioral and psychological symptoms, map the symptoms
	☐Yes ☐No In case of nutritional difficulties, perform nutritional mapping
	☐Yes ☐No In case of acute confusion (delirium), map the synptoms and contact a doctor
**Agree upon a time and place for the case conference: TID administrator/manager**

**Table 2:** Agenda and timeframe for the case conferences

**Agenda for guided reflection meeting (case conference), approximately 1 h**	
Activity	Preparation: Convene a meeting and prepare a meeting room with a blackboard or similar facilities (projector, if available). Check that a flip pad and markers are available. As many as possible from the home care service staff should attend the conference. The leading registered nurse and the GP should attend the conference, if possible
	1. Status Report: Personal history and main points from the patient’s medical record are presented, 10 min. Decide in advance who should prepare and present the patient’s personal history and the main points from the medical record
	2. Create a problem list, approximately 5 min
	3. Prioritise problems from the list, approximately 5 min
	4. Draw a five − column table on the whiteboard that includes facts – interpretations (thoughts)—emotions – actions – evaluation
	5. Describe facts from the registration and assessment phase one problem at a time, approximately 10 min
	6. Suggest interpretations – guided discovery – discuss and reflect on the interpretations, approximately 10 min
	7. Describe any emotions experienced by the staff with interpretations by the staff, approximately 10 min
	8. Suggest SMART (Specific, Measurable, Achievable, Relevant, Time-bound) actions based on the interpretations and decide how and when to perform an evaluation of the actions, approximately 10 min
	9. Summarise interpretations and actions and close the meeting, approximately 5 min

One specialist registered nurse from the education and training team will attend and supervise the TIME administrators’ first case conference on their first patient in their municipality. For the remainder of the intervention and for the other patients included in the trial, the TIME administrators and staff will carry out the intervention independently.

### Criteria for discontinuing or modifying allocated interventions {11b}

Participation in the project is voluntary. The participant can withdraw consent at any time and without giving any reason. This will have no consequences for further home care services. We have not established any other criteria for modifying the allocated intervention for any given trial participant.

Members of the research team at the AFS research centre will contact the local project nurses in each municipality twice with 2-month intervals. A structured telephone interview with a checklist that consists of the core components of TIME (i.e. the assessment phase, case conferences, the actions taken and systematic evaluations of these actions) will be carried out.

### Relevant concomitant care permitted or prohibited during the trial {11d}

The services and treatment measures provided are generally recognised care and treatment. The patients in the control group will receive care and treatment as usual. We have no other criteria for concomitant care permitted or prohibited during the trial.

### Provisions for post-trial care {30}

Not applicable, as there is no post trial care.

### Outcomes {12}

#### Baseline data and primary and secondary outcome measures

The primary outcome of the trial is the difference in the change between the intervention and control groups in individual goal achievement to resolve or alleviate the challenges regarding crises between baseline and 3 months using the PGSI (scale of 1–10) [40]. We chose this primary outcome because there is a need for a targeted outcome that comprises the variability in a heterogeneous population. It is very unlikely that a participant would be harmed due to participation in the RCT; therefore, it is not deemed necessary to have a harm outcome in the study. In the RCT, no new experimental treatments for the patients will be introduced, and care and all treatment actions will rely on recommended national care and treatment guidelines.

The secondary outcomes are the differences in the change between the intervention and control groups in the PGSI scale at 6 months, in neuropsychiatric symptoms (NPSs) measured by the Neuropsychiatric Inventory (NPI-NH) [43], quality of life measured by the Quality of Life in Late-Stage Dementia scale (QUALID) [44, 45], distress perceived by the next of kin measured by the Relative Stress Scale [46], rejection of care measured by the Minimum Data Set [47], activities of daily living assessed with the Physical Self-Maintenance Scale (PSMS) [41], prescribed medications collected from the medical records [48], frailty measured with the Clinical Frailty Scale (CFS) [39], institutionalisation at 3 and 6 months, and pain and discomfort assessed by the EQ-5D questionnaire [49] at 6 months. All these questionnaires have been proven to have acceptable validity and reliability. The trial will also collect data to be used as covariates in the RCT and to describe the sample of participants. These data will be collected with questionnaires answered by the staff in home care services:

- Age (covariate in the RCT), sex, level of education and employment status, marital status, living conditions (living alone or with someone)
- Hours a week and type of home care service
- Relation to next of kin (e.g. next of kin and how often they meet)
- Physical health measured with the General Medical Health Rating Scale (GMHR) [50]
- Cognitive function assessed by the Clinical Dementia Rating Scale (CDR) [51] (covariate in the RCT).

### Participant timeline {13}

The study timeline for enrolment, intervention, and assessment is described in Table 3. In addition, description of the questionnaires and the time points at which they are administered is provided in Table 4.

**Table 3:** Enrolment, intervention, and assessment schedule

Timepoint^a^		Study period			
	Enrolment	Pre-allocation	Allocation	Post-allocation	
	− t0	t1	0	t2	t3
Enrolment					
Eligibility	X				
Informed consent	X				
Allocation/randomisation			X		
Interventions					
Joint education and training		X			
Intervention group				X	X
Control group				X	X
Assessments					
Baseline		X			
3 months				X	
6 months					X

A detailed overview of the assessments is presented in the “Outcomes {12}” section

^a^t1, baseline; t2, 3 months after baseline; t3, 6 months after baseline

**Table 4:** Overview of data collection with primary and secondary outcome measures

Data collected	Interviewers	Respondents	Baseline	Three months	Six months
Characteristics of the participants					
Age^a^, sex, level of education and employment status, marital status and living conditions	Data assessors from AFS^b^	Staff members in home care services	X		
Hours a week and type of service from the home care service	Data assessors from AFS	Staff members in home care services	X		
Number of visits per day from the home care service	Data assessors from AFS	Staff members in home care services	X		
Relation to next of kin (e.g. next of kin and how often they meet)	Data assessors from AFS	Staff members in home care services	X		
Diseases (known diagnosis)	Data assessors from AFS	Staff members in home care services	X		
Frailty measured with the Clinical Frailty Scale (CFS)^c^	Data assessors from AFS	Staff members in home care services	X		X
Physical health measured with the General Medical Health Rating Scale (GMHR)^d^	Data assessors from AFS	Staff members in home care services	X		
Cognitive function measured with the Clinical Dementia Rating Scale (CDR)^e^	Data assessors from AFS	Staff members in home care services	X		
Primary outcome					
PRACTIC Goal Setting Interview (PGSI)^f^	Data assessors from AFS	Patient, next of kin and staff members in home care services	X	X	X
Secondary outcomes					
Medication from medical records	Data assessors from AFS	Staff members in home care services	X	X	X
Rejection of care evaluated by the Minimum Data Set (MDS)^g^	Data assessors from AFS	Staff members in home care services	X	X	X
Neuropsychiatric Inventory Nursing Home version (NPI-NH)^h^	Data assessors from AFS	Staff members in home care services	X	X	X
Activities of daily living assessed with the Physical Self-Maintenance Scale (PSMS)^i^	Data assessors from AFS	Staff members in home care services	X	X	X
The EQ-5D questionnaire^j^ to evaluate pain and discomfort	Data assessors from AFS	Patient	X		X
Quality of Life in Late-Stage Dementia scale (QUALID)^k^	Data assessors from AFS	Staff members in home care services	X	X	X
RSS (Relative Stress Scale)^l^ Next of kin	Data assessors from AFS	Next of kin	X	X	X

Data describing the municipalities and the organisation of the home care services will be assessed in a separate process evaluation study of the PRACTIC project [29]

^a^Age is to be used as a covariate in the RCT

^b^*AFS*, Research Centre for Age-related Functional Decline and Disease; *AFS*, Innlandet Hospital Trust

^c^Clinical Frailty Scale (CFS) [39]

^d^General Medical Health Rating Scale (GMHR) [50]

^e^Clinical Dementia Rating Scale (CDR) is to be used as a covariate in the RCT [51]

^f^PRACTIC Goal Setting Interview (PGSI) [40]

^g^Minimum Data Set (MDS) [47]

^h^Neuropsychiatric Inventory (NPI-NH) [43]

^i^Physical Self-Maintenance Scale (PSMS) [41]

^j^EQ-5D questionnaire [49]

^k^Quality of Life in Late-Stage Dementia scale (QUALID) [44, 45]

^l^Relative Stress Scale [46]

### Sample size {14}

#### Sample size calculation based on the primary outcome

The proposed sample of 150 participants is based on a power calculation with clusters of approximately five participants from each of the 30 municipalities. Based on a previous trial, a minimal clinically important average difference on the PGSI scale between participants in the intervention and control groups was set to 2 points with a standard deviation (SD) for change of 2.83 in each group [40]. To observe a statistically significant difference with a power of 80%, an intracluster (municipality) correlation coefficient (ICC) of 10%, and an estimated attrition rate of 25% for the primary outcome at 3 months, we will need approximately 150 participants. We assume a high attrition rate since the participants are at high risk for acute institutionalisation (see the inclusion criteria).

### Recruitment {15}

The local project nurses (See: Who will take informed consent? {26a}) will support the research team in the recruitment of participants. Care providers for the intervention will, however, be the permanent staff in the home service units. Each municipality must recruit approximately five patients, and the project nurses have one week to include participants in the study. These nurses will know the patient well and assess whether the inclusion criteria are fulfilled for each patient for participation in the study. The project nurses will ask eligible patients if they wish to participate when they visit the patient in their home. Each IM and CM will be reimbursed 900 EUR for the time spent in this recruitment process.

## Assignment of interventions: allocation

### Sequence generation {16a}

This trial will be conducted as a cluster randomised controlled study, where each municipality represents a cluster. The clusters will be randomised to either receive the locally adapted TIME intervention (the intervention group) or care and treatment as usual (the control group). The intervention is a biopsychosocial intervention that involves the entire interdisciplinary team and staff in the home care service units of the participating municipalities to optimise the approach towards a group of patients in the municipalities. Thus, without cluster randomisation, the study runs the risk of implementing all or parts of the intervention model among individual control patients in the same municipalities [52].

The municipalities will be stratified by size into three blocks to ensure approximately the same number of patients in the two trial arms. These blocks are (1) small municipalities, (2) medium-sized municipalities, and (3) large municipalities. The municipalities within each block will be randomly assigned to either the intervention group or the control group. A statistician independent of the project management team and the municipalities will perform a computer-generated randomisation procedure.

### Concealment mechanism {16b}

The randomisation results for the municipalities will be sent from the independent statistician by email to a researcher (AV) in the research team by an encrypted file and a connection key. Only this researcher and the research team will know the randomisation results.

### Implementation {16c}

A statistician independent of the project management team and the municipalities will perform the randomisation by the use of a computer procedure. The project management team will provide the home care services in the municipalities with the randomisation and allocation results immediately after the randomisation procedure. The intervention will start with the educational sessions (11a) within 1 to 2 weeks after randomisation.

## Assignment of interventions: blinding

### Who will be blinded {17a}

The baseline assessments, before randomisation, will be performed by the same data assessors by visiting the participants in their homes and interviewing them, the next of kin, and the staff members who know the patient best. The data assessors in the study will be blinded to whether the municipality is allocated to the intervention group or the control group at the assessments at 3 months and 6 months. These data assessments will be performed by telephone by interviewing the staff 3 months and 6 months after baseline. The statistical data analyst will also be blinded to the allocation results. After baseline and the allocation process, the home care services, the training group, and the patients cannot be blinded.

### Procedure for unblinding if needed {17b}

Unblinding during the study will not be permitted, and the data assessors will be blinded to the randomisation result.

## Data collection and management

### Plans for assessment and collection of outcomes {18a}

The data assessors are specially trained nurses from the project’s research centre who are not affiliated with the municipalities. All data assessors are registered nurses with substantial experience and formal training in the use of the assessment scales, and they will attend a 1-day course about the use of the assessment scales before the start of the trial. A description of the questionnaires, including data assessors, respondents, and the time points at which they are administered, is provided in Table 4.

### Plans to promote participant retention and complete follow-up {18b}

The participants will be assessed at three time points, at baseline and at 3 and 6 months, during the 6-month RCT. The participants in the intervention group will also be followed up by phone by the local project nurses using a checklist for adherence to the study protocol 2 and 4 months from baseline. For all participants who withdraw from the study before the 6-month follow-up, the date and reason for discontinuation will be registered by the local project nurses and reported to the research team.

### Data management {19}

The data protection official, Innlandet Hospital Trust, has approved the applications for data entry and security. The collected data from the sampled patients will be deidentified and stored on a secured research server at Innlandet Hospital Trust.

### Confidentiality {27}

Specially trained nurses (data assessors) from the project’s research centre who are not affiliated with the municipalities will travel to the municipality to assess baseline data using a paper case report form (CRF). Data will also be assessed by the same data assessors at 3 and 6 months using the CRF, such as telephone interviews with nurses from the municipality. The data assessors will deliver the CRFs directly to the researchers in the project group at the research centre when they have been completed. The data will then be scanned and transferred into SPSS (Statistical Package for the Social Sciences [53]) at the project’s research centre. The SPSS file will be stored on a secure server at Innlandet Hospital Trust. The data will be kept for 5 years after the end of the project for control reasons.

### Plans for collection, laboratory evaluation, and storage of biological specimens for genetic or molecular analysis in this trial/future use {33}

N/A. Collection, laboratory evaluation, and storage of biological samples for genetic or molecular analysis is not applicable in this study.

## Statistical methods

### Statistical methods for primary and secondary outcomes {20a}

#### Data processing and statistical analysis of quantitative data

The data will be presented as frequencies and percentages for categorical variables and means with standard deviations for continuous variables. The normality of the continuous variables will be assessed graphically. If necessary, skewed data will be transformed. The analyses for primary and secondary outcomes will be adjusted for baseline PGSI scores, baseline severity of dementia (Clinical Dementia Rating), and age of the participants. Differences in the changes in outcomes between the intervention group and the control group will be assessed by a linear mixed model with fixed effects for time component and group and the interaction between the two. A significant interaction will imply the differences in change between the groups. Random effects for patients nested within municipalities and slopes (if significant) will be included in the model. Individual time point contrasts will be derived within each group at each time point with the corresponding 95% confidence intervals and *p*-values. The linear mixed model correctly adjusts estimates for intracluster correlations as well as for intraindividual correlations due to repeated measurements over time.

The analyses for primary and secondary outcomes will be adjusted for differences in:

- Baseline PGSI scores
- Baseline severity of dementia (Clinical Dementia Rating Scale, CDR) [51]
- Age

Other descriptive analysis to further describe the sample of participants:

f) Sex, level of education, and employment status, marital status, living conditions (living alone or with someone)
g) Hours a week and type of home care service
h) Relation to next of kin (e.g. next of kin and how often they meet)
i) Physical health measured with the General Medical Health Rating Scale (GMHR) [50]

### Interim analyses {21b}

N/A. No preliminary analyses will be performed, and we have not developed any stopping guidelines. All treatment measures developed and implemented for the patients in the intervention group are regular and known clinically accepted procedures and care, with no new experimental measures or treatments.

### Methods for additional analyses (e.g. subgroup analyses) {20b}

N/A. There are currently no planned additional analyses.

### Methods in analysis to handle protocol non-adherence and any statistical methods to handle missing data {20c}

To handle protocol nonadherence, the analysis will be performed as an intention-to-treat analysis. The linear mixed model handles unbalanced data by allowing the inclusion of all available information, including dropouts and missing data.

### Plans to give access to the full protocol, participant-level data, and statistical code {31c}

The last version of the full protocol and the datasets will be made available on the project’s website: www.practic.no. Participant-level data and the statistical code will only be accessible to the central research team.

## Oversight and monitoring

### Composition of the coordinating centre and trial steering committee {5d}

This study is a part of the larger PRACTIC study. The project is owned by the Research Centre for Age-Related Functional Decline and Disease (AFS), Innlandet Hospital Trust, Ottestad, Norway. The core research group with the project leader, work package leaders, postdocs, PhD students, and a project coordinator will have day-to-day responsibility for the project. A project group comprising the core research group and other research staff will have regular meetings once a month to discuss the project. The PhD students and research staff will collaborate with participating municipalities in the project and have regular dialogue. The PhD students are responsible for coordinating and collaborating with the training group for educational sessions during the intervention and the data assessor group throughout the RCT.

The project has organised two reference groups, one with end-users (patients and next of kin) and one with staff from home care services, including GPs. The reference groups will meet with the central project group regularly during the project period. The design of the project will enable close cooperation with end-users and stakeholders in the municipalities through the establishment of local project groups in each municipality.

### Composition of the data monitoring committee, its role and reporting structure {21a}

This trial does not require periodic inspections of cumulative outcome data. The study involves little risk for the participants, so there is no need for periodic inspection of accumulating outcome data by a formal committee such as a data monitoring committee (DMC).

### Adverse event reporting and harms {22}

The data protection official, Innlandet Hospital Trust, has approved applications for the trial. If participants have questions about privacy in the project, they can contact the institution’s data protection officer. If any adverse events occur, this will be reported to the data protection officer.

In this study, no new or experimental treatments will be provided to the participants, and they will receive only traditional health care and assistance for their needs. The intervention mainly comprises an introduction of a more person-centred way of working for the staff that is very unlikely to cause harm to the patients. The principal investigator (PI) for the study is responsible for the daily operation of the study, and the participants or their next of kin can contact the project leader in any cases of harm or questions pertaining the study.

### Frequency and plans for auditing trial conduct {23}

N/A. There is no requirement for the frequency of or procedures for auditing the conduct of the trial.

### Plans for communicating important protocol amendments to relevant parties (e.g. trial participants, ethical committees) {25}

In the event of important protocol changes, a change notification will be sent to the data protection officer, Innlandet Hospital Trust.

### Dissemination plans {31a}

The datasets supporting the conclusions of this article will be available on the website for the PRACTIC study. Three papers will be published in international peer-reviewed open access journals. In addition, the results from this study will be presented orally at national and international congresses. The results of the project will also be communicated to participants in the train the trainer course in TIME.

## Discussion

Through interventions customised to the patients and targeting their next of kin, the patients’ social context, and health care services, we hypothesise that crises can be prevented and resolved with the use of an adapted TIME intervention. Home care services can be described as a complex organisation and consist of many different units and functions involved in the services [7]. There is a need for a holistic understanding. This project aims to test the effectiveness of an adapted version of a biopsychosocial person-centred model (TIME) to prevent and resolve crises for frail residents receiving home care. The strength of the model is that it was developed over a period of several years and has been used in clinical practice for other complex problems, such as nutritional deficiencies, multimorbidity, and general loss of function [30]. A pilot study demonstrated the feasibility of the model in home care services [33].

One of the challenges of including patients who receive home care services in trials is that they represent a heterogeneous group and vary substantially in function, age, and living conditions as well as illness and diseases [8]. Many of these patients have several chronic conditions (multimorbidity) [4], and most frail individuals are multimorbid [11]. Based on this, we have chosen the inclusion criteria for the study to be quite broad to make the trial more pragmatic [54]. The advantage is that the TIME model is used in situations and challenges where the staff deems it necessary to work in a structured way. A literature review of the definition of crises in dementia care noted that patient perceptions, their next of kin, and health care staff are interrelated and depend on the type of crisis stressors and where the crises take place [15]. This strengthens the model’s properties in a home care context and the need for a holistic approach to the crisis.

The study is a cluster randomised controlled trial (RCT), which is the gold standard for testing the effectiveness of a certain type of treatment or model. Furthermore, we have combined this design with participatory action research (PAR) to allow for local adaptations of the intervention to the local context and needs [23, 24]. A flexible intervention is an important factor for the measures to be effective and increase their applicability in practice. The study by Lichtwarck et al. [28] showed that even though TIME is a complex intervention, it did not require major changes in the organisations’ structures or routines, and the implementation costs were estimated to be low. For complex interventions in complex settings, flexibility within certain limits is a success factor. According to Hawe et al. [24], it is the function and processes of the intervention that should be standardised, not details in the components of the intervention.

Our study design has some limitations. We do not require a specific diagnosis as an inclusion criterion; instead, we will include patients who are frail, defined as a CFS score from 5 to 8, and considered by the home care services to be in an unstable situation, an imminent crisis. This will necessarily result in a heterogeneous sample for the trial and may jeopardise the possibility of demonstrating any effectiveness of the intervention. On the other hand, the sample will mimic a real-world population receiving home care services and add to the pragmatic character of the trial [54]. Regardless of their diagnosis, the patients are frail and require comprehensive assessments and follow-up from home care services.

Since the participants in the trial represent a heterogeneous group, there is a need for a primary outcome that accounts for this variation between patients. The treatment measures taken during the trial and the outcomes will necessarily vary from patient to patient [26]. The PGSI has the potential to address this heterogeneity and will guide the development of measures during the intervention. We will therefore be able to measure potential differences in change in goal achievement measured with the PGSI between IMs and CMs 3 months from baseline. The PGSI is a Norwegian adapted version of the BGSI [40], a validated tool that has been simplified and adapted to be used by home care services. We will shortly after the trial perform a qualitative content validation study of the PGSI. This goal-setting interview is also considered clinically important and can be used by home care services after the intervention has been completed. A limitation may be that introducing the PGSI as the primary outcome in the trial will affect all municipalities, including the control municipalities. The PGSI is a minor intervention in itself and may reduce the possibility of demonstrating a significant difference between IMs and CMs pertaining to the primary outcome. On the other hand, introducing the PGSI in both groups may reduce the risk of observer bias, a bias that will often be present in a trial that is only single-blinded. By introducing the PGSI for the staff in the CMs, they will have a sense of being part of an intervention [55].

### Potential impact of the proposed research

Due to the multimorbidity and diversity of the patients receiving home care services, care interventions can hardly be introduced as standardised solutions based on single diagnoses but should be implemented through holistic approaches embracing multimorbidity and different functional impairments. The PRACTIC study will enhance innovation in the development of new knowledge and the development of a new biopsychosocial approach towards each patient. This process will be adapted to local structural conditions. The project is likely to enforce systematic cooperation between home care services and GPs, where diagnostic work-up and follow-up will be one of the most important tasks. For home care services, this means a cultural change from a mainly task-oriented service based on support for the activities of daily life to an interdisciplinary assessment and follow-up of functional impairment and diseases for these patients. If successful, because of the trial’s pragmatic character, the intervention can easily be implemented in health care services with minimal extra resources. Improving the approaches to crises may also reduce the use of specialist health care services.

## Trial status

In accordance with the trial protocol version 9, dated 30 June 2023, the cluster randomised trial started with inclusion of the first patients on the 6 January 2023, and the last patients will be included on the 26 October 2023.

The last visit to the patients is scheduled for April 12, 2024. The reason for the delayed submission is the comprehensive recruitment process that involves both participating municipalities and study participants. An unexpected lack of resources pertaining enough staff in the municipalities to support the recruitment has affected the ability to submit the protocol earlier. Most of the efforts in the research team has been directed towards optimising and supporting this recruitment process. The recruitment was performed through multiple stages during the RCT in 2023. Since the municipalities involved in the project have varying start dates for the RCT, the recruitment of the last patient was on 20 October 2023. The final visit to the patients is scheduled for April 12, 2024.

### Supplementary Information

**Additional file 1.** Consent form for the patient and their next of kin regarding participation.

**Additional file 2.** English translation of the consent form.

## Data Availability

The template for the case report forms (CRFs) is available on the PRACTIC website, www.practic.no. Data collected in the study are not publicly available. Data are available upon reasonable request to the members of the research team at the AFS research centre.

## Abbreviations

AFS: Research Centre for Age-related Functional Decline and Disease, Innlandet Hospital Trust
BPSD: Behavioural and Psychological Symptoms in Dementia
CBT: Cognitive behavioural therapy
CDR: Clinical Dementia Rating Scale
CFS: Clinical Frailty Scale
CMs: Control municipalities
EQ-5D-5L: European Quality of Life Scale
GMHR: General Medical Health Rating Scale
GPs: General practitioners
IMs: Intervention municipalities
MOBID-2: Mobilisation-Observation-Behaviour-Intensity-Dementia Scale
MDS: Minimum Data Set
NPI-NH: Neuropsychiatric Inventory-Nursing Home Version
NPSs: Neuropsychiatric symptoms
PGSI: PRACTIC Goal Setting Interview
PRACTIC: Preventing and Approaching Crises for Frail Community-dwelling Patients Through Innovative Care
QUALID: Quality of Life in Late-stage Dementia
RSS: Relative Stress Scale
SMART: Specific, Measurable, Actual, Realistic, Time-bound
SPSS: Statistical Package for the Social Sciences
TIME: Targeted Interdisciplinary Model for Evaluation and Treatment of Neuropsychiatric Symptoms
VAS: Visual analogue scale
