# Correction: PReventing and Approaching Crises for frail community-dwelling patients Through Innovative Care (PRACTIC): protocol for an effectiveness cluster randomised controlled trial

**DOI:** 10.1186/s13063-024-08257-9

**Published:** 2024-06-27

**Authors:** Anette Væringstad, Ellen Thea Gjelseth Dalbak, Daniela Holle, Janne Myhre, Øyvind Kirkevold, Sverre Bergh, Bjørn Lichtwarck

**Affiliations:** 1 The Research Centre for Age-Related Functional Decline and Disease, Innlandet Hospital Trust, Ottestad, Norway; 2 Department of Health, Care and Nursing, Faculty of Medicine NTNU, Norwegian University of Science and Technology, Gjøvik, Norway; 3 Department of Nursing Science, University of Applied Sciences (HS Gesundheit), Bochum, Germany; 4 Faculty of Social and Health Sciences, Inland Norway University of Applied Sciences (INN University), Elverum, Norway; 5 The Norwegian National Centre for Ageing and Health, Vestfold Hospital Trust, Vestfold, Norway

**Correction** **:** **Trials 25, 304 (2024)**

**https://doi.org/10.1186/s13063-024-08117-6**

Following the publication of the original article [1], we have been notified that Figure 1 was incomplete.

Originally published Figure 1:


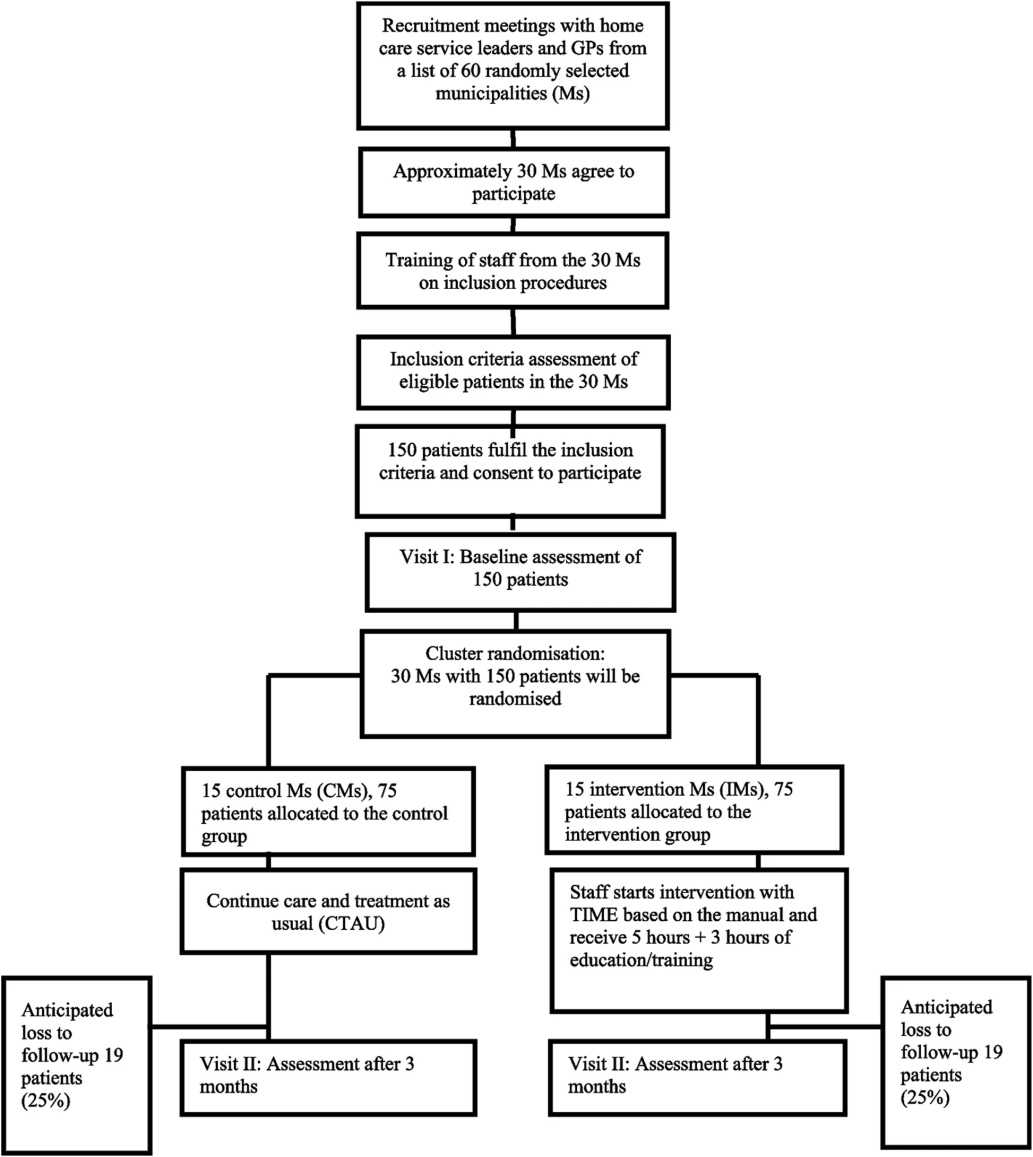


Corrected Figure 1:

**Fig. 1:**
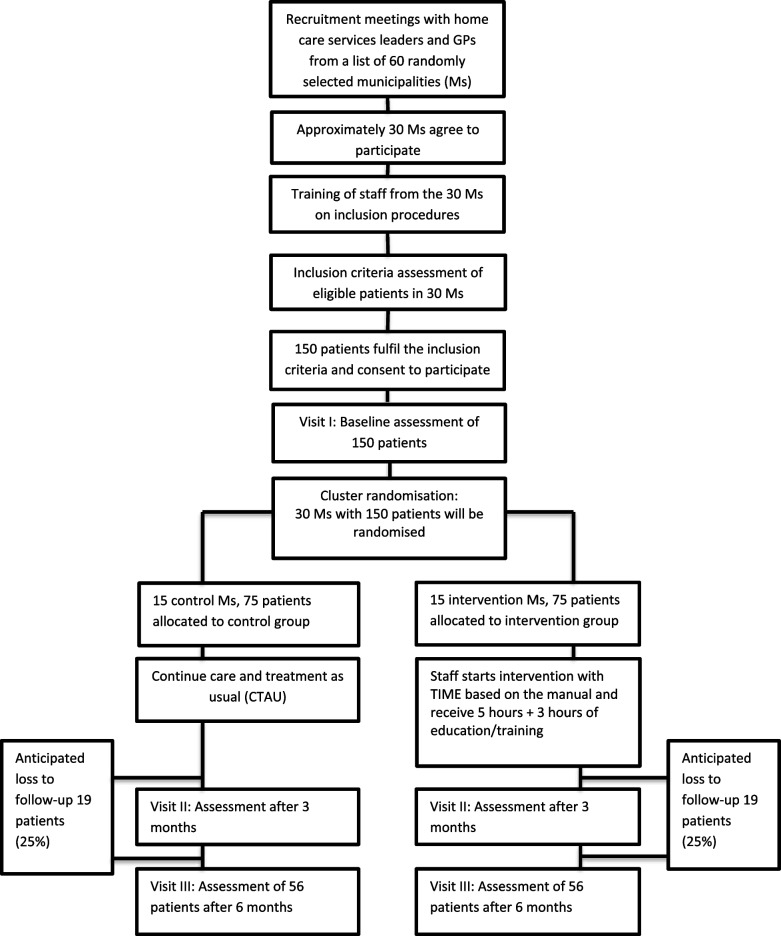
The PRACTIC trial: Flowchart of the clusters and patients throughout the phases of the trial

The original article has been corrected.
