# Supplementary material for: PReventing and Approaching Crises for frail community-dwelling patients Through Innovative Care (PRACTIC): protocol for an effectiveness cluster randomised controlled trial
Source: Trials. 2024 May 6;25:304. doi: 10.1186/s13063-024-08117-6 (PMC11075302; doi:10.1186/s13063-024-08117-6)
Supplement: Supplementary file 1 — Additional file 1. Consent form for the patient and their next of kin regarding participation. [file 13063_2024_8117_MOESM1_ESM.pdf]

Sykehuset Innlandet HF
Divisjon Psykisk helsevern

AFS

Appendix 1

REQUEST FOR PARTICIPATION IN THE RESEARCH PROJECT PRACTIC

THE PURPOSE OF THE PROJECT AND WHY YOU ARE BEING ASKED

This is a request for your participation in a research project called PRACTIC, which focuses on preventing and addressing crises in vulnerable individuals who live at home. The purpose of the project is to develop and adapt a new model called the "TIME model" for preventing and resolving crises. The TIME model is an assessment and reflection model, a working tool for home care staff. The model provides a more systematic approach to assessing and treating symptoms in complex conditions.

You have been selected for this project because you are in need of home care services. The municipality you live in is participating in the PRACTIC project, and you are being asked to participate because we believe you can provide valuable knowledge regarding the testing of the model. The Research Centre for Age-related Functional Decline and Disease, (AFS), Innlandet Hospital Trust, is responsible for the project and all the information collected.

WHAT DOES THE PROJECT INVOLVE?

In the project, one group of municipalities will receive training in the TIME model, while another group of municipalities will not receive training and will serve as control municipalities. Neither you nor the municipality will know whether it will be a control municipality or a municipality receiving training in the TIME model until the project begins. If the municipality you live in receives training in the TIME model, it will involve a comprehensive assessment and an interdisciplinary reflection meeting regarding your situation. In the reflection meeting, home care staff will develop tailored measures to address your needs. Regardless of whether the municipality you live in becomes a control municipality or receives training in the TIME model, we will need health information about you.

- There will be an interview at your home, preferably with your closest relative. The interview will include questions about your personal goals and self-assessed health. The interview will take approximately 45 minutes, and you will be interviewed three times over 6 months.
- We will gather information from the home care staff who know you well. The staff will provide us with health information related to possible mental (e.g., depression, anxiety, memory) and physical illnesses/symptoms (e.g., pain, functional level). We will record this information using various assessment tools.
- The home care staff may use information from your medical records to describe your use of services, prescribed medications, and current diagnoses. Only staff who have daily access to your medical records will be able to access this information. The information will be used to provide a solid basis for assessing your condition and planning your service to best meet your needs. The service provided is standard recognized care and treatment.

Appendix 1

POSSIBLE BENEFITS AND DRAWBACKS

Experiences from the project may lead to the development of a customized version of the TIME model as a tool for home care services, which could contribute to improved quality of care and treatment for patients receiving home care services. Participating in the project may result in better observation and follow-up of your needs, leading to services that are better tailored to your needs. A disadvantage of participating in the project is that you and your relatives will need to allocate time to answer questions about your health.

WHAT HAPPENS TO YOUR INFORMATION?

The information recorded about you will only be used as described for the purpose of the project and without direct identifying information. A code will link you to your information through a name list. Only approved personnel associated with the project will have access to the name list and be able to trace it back to you. It will not be possible to identify you in the project results when they are published. You have the right to access the information recorded about you and the right to correct any errors in the information that has been recorded. If you wish to access this information, you have the right to receive the information within 30 days. You can file a complaint about the handling of your information with the Data Protection Authority and the institution's Data Protection Officer. The information will be stored on a secure research server at Innlandet Hospital Trust. The information will be presented in a scientific research article. It will not be possible to identify you in the project results when they are published. The project leader is responsible for the daily operation of the research project and for ensuring that your information is handled securely. The information about you will be anonymized. The information about you will be kept for five years after the project ends for control purposes.

VOLUNTARY PARTICIPATION AND THE RIGHT TO WITHDRAW CONSENT

Participation in the project is voluntary. If you wish to participate, you will sign the consent form on the last page. You can withdraw your consent at any time without providing a reason. This will not have any consequences for your ongoing treatment. If you withdraw from the project, you can request that collected information be deleted, unless the information has already been included in analyses or used in scientific publications.

If you later wish to withdraw or have questions about the project, you can contact the project leader (see contact information below).

INSURANCE

As a patient in the Norwegian healthcare system, you are covered by the Patient Injury Act. Participation in this research project will be covered under the same insurance. All information about you is subject to new privacy legislation and is legally regulated by the EU's General Data Protection Regulation.

APPROVAL

The project is registered and approved by the Data Protection Officer at Innlandet Hospital Trust, case number: 23928741. Project leader Bjørn Lichtwarck is responsible for data protection in the project. We process the information based on your consent.

Appendix 1

CONTACT INFORMATION

If you have questions about the project or wish to withdraw from participation, you can contact Bjørn Lichtwarck, mobile 975 23 048, email bjorn.lichtwarck@sykehuset-innlandet.no, or research fellow Anette Væringstad, mobile 995 40 470, anette.vaeringstad@sykehuset-innlandet.no.

If you have questions about data protection in the project, you can contact the data protection officer at the institution: personvernombud@siv.no or Personvernombudet@sykehuset-innlandet.no. The Data Protection Authority's email address is postkasse@datatilsynet.no.

Appendix 1

CONSENT TO PARTICIPATE IN THE PRACTIC PROJECT

I AM WILLING TO PARTICIPATE IN THE PROJECT

Location and date | Participant's signature

Participant's name (printed)

AUTHORIZED CONSENT

As the closest relative of _ (Full name), I consent to her/his participation in the project.

Location and date | Relative's signature

Relative's phone number | Relative's name (printed)

I CONFIRM HAVING PROVIDED INFORMATION ABOUT THE PROJECT

Location and date | Signature

Role in the project
