# Supplementary material for: PReventing and Approaching Crises for frail community-dwelling patients Through Innovative Care (PRACTIC): protocol for an effectiveness cluster randomised controlled trial
Source: Trials. 2024 May 6;25:304. doi: 10.1186/s13063-024-08117-6 (PMC11075302; doi:10.1186/s13063-024-08117-6)
Supplement: Supplementary file 2 — Additional file 2. English translation of the consent form. [file 13063_2024_8117_MOESM2_ESM.pdf]

Appendix 2

REQUEST FOR PARTICIPATION IN THE RESEARCH PROJECT PRACTIC

INFORMATION LETTER TO FAMILY MEMBERS ABOUT – HEALTH ECONOMICS AND CAREGIVER BURDEN QUESTIONNAIRE

THE PURPOSE OF THE PROJECT AND WHY YOU ARE ASKED

This is a question for you as a family member of someone participating in a research project called PRACTIC, which focuses on the prevention and approach to crises among vulnerable individuals living at home. The purpose of the project is to develop and adapt a new model (the TIME model) for preventing and resolving crises.

The TIME model is an assessment and reflection model, a tool for home care staff. The model provides a more systematic approach to assessing and treating symptoms in complex conditions. You are being asked to participate in this study because you are a family member of the person receives home care services. The Research Centre for Age-related Functional Decline and Disease, (AFS), Innlandet Hospital Trust, is responsible for the project and all the information collected. The results of the project will be published as scientific articles. All information about you will be anonymized, meaning that no participants will be identifiable in these publications.

WHAT DOES THE PROJECT INVOLVE?

In the project, a group of municipalities will receive training in the TIME model, while another group of municipalities will not receive training in the TIME model and will serve as control municipalities. Neither you nor the municipality will know before the project starts whether the municipality you live in will be a control municipality or a municipality receiving TIME model training. Regardless of whether the municipality you reside in becomes a control municipality or a municipality receiving TIME model training, we need some information from you.

Home care staff will arrange with you for three calls over a period of 6 months (at the beginning of the project, at 12 weeks, and at 24 weeks) when a person from Innlandet Hospital Trust calls you to ask questions related to health economics and the burden of being a caregiver. You will receive the questions in advance from the home care service so that you can be more prepared. The information will be used to provide a good basis for the assessment of the person you are a caregiver for and to plan the services to best meet the person's needs. The services provided are standard recognized treatment.

POTENTIAL BENEFITS AND DRAWBACKS

Experiences from the project may lead to the development of a customized version of the TIME model as a tool for home care services. The project will contribute to ensure that the person you are a caregiver for receives

services better tailored to their needs. The drawback of participating in the project is that, as a family member, you will need to allocate time to answer questions about health economics and caregiver burden.

WHAT HAPPENS TO YOUR INFORMATION?

The information recorded about you will only be used as described in the project's purpose, and you will not be directly identifiable in the data. A code links you to your information through a name list. Only approved personnel associated with the project will have access to the name list and be able to identify you. It will not be possible to identify you in the project's results when they are published. You have the right to access the information recorded about you and the right to have any errors in the information corrected. If you wish to access this information, you are entitled to receive it within 30 days. The project leader is responsible for the day-to-day operation of the research project and for ensuring that your information is handled securely. Your information will be anonymized. Your information will be retained for five years after the project ends for control purposes.

VOLUNTARY PARTICIPATION AND THE POSSIBILITY TO WITHDRAW CONSENT

Participation in the project is voluntary. If you choose to participate, you will sign the consent form on the last page. You can withdraw your consent at any time without providing a reason. This will not have any consequences for you or the person you are a caregiver for. If you withdraw from the project, you can request that the collected information be deleted unless the information has already been included in analyses or used in scientific publications. If you later wish to withdraw or have questions about the project, you can contact the project leader (see contact information below).

INSURANCE

As a participant in this research project, you will not receive treatment, so insurance is not considered necessary. All information about you is subject to new data protection laws and is legally regulated by the EU General Data Protection Regulation.

APPROVAL

The project is registered and approved by the data protection officer at Innlandet Hospital Trust, case number: 23928741. Project leader Bjørn Lichtwarck is responsible for data protection in the project. We process your information based on your consent.

CONTACT INFORMATION

If you have questions about the project or wish to withdraw from participation, you can contact Bjørn Lichtwarck, mobile 975 23 048, email bjorn.lichtwarck@sykehuset-innlandet.no , or research fellow Anette Væringstad, mobile 995 40 470, anette.vaeringstad@sykehuset-innlandet.no

If you have questions about data protection in the project, you can contact the data protection officer at the institution: personvernombud@siv.no or Personvernombudet@sykehuset-innlandet.no .The Data Protection Authority's email address is postkasse@datatilsynet.no

Sykehuset Innlandet HF
Divisjon Psykisk helsevern

AFS

Appendix 2

CONSENT TO PARTICIPATE IN THE PRACTIC PROJECT

I AM WILLING TO PARTICIPATE IN THE PROJECT

As a family member of ________________________________ (patient's name in block letters), I am willing to participate in the research project.

Location and date

Participant's signature

Family member's phone number

Participant's name in printed letters

I CONFIRM THAT I HAVE PROVIDED INFORMATION ABOUT THE PROJECT

Location and date

Signature

Role in the project
